# Too much, too soon? Two Swedish case studies of short-term deadwood recruitment in riparian buffers

**DOI:** 10.1007/s13280-022-01793-1

**Published:** 2022-10-08

**Authors:** Lenka Kuglerová, Gustaf Nilsson, Eliza Maher Hasselquist

**Affiliations:** Department of Forest Ecology and Management, Swedish University of Agricultural Sciences, Skogsmarksgränd 17, 907 36 Umeå, Sweden

**Keywords:** Ecological functions, Headwaters, Large wood, Riparian buffer, Sustainable forest management, Trollberget experiment

## Abstract

Forested riparian buffers are retained along streams during forest harvest to maintain a number of ecological functions. In this paper, we examine how recently established riparian buffers along northern Swedish streams provide deadwood, a key objective for riparian buffer management in Sweden. We used observational and experimental data to show that the investigated buffers provided large volumes of deadwood to streams and riparian zones shortly after their establishment, likely jeopardizing continued recruitment over the long term. Deadwood volume decreased with increasing buffer width, and the narrowest buffers tended to blow down completely. Wider buffers (~ 15 m) provided similar volumes of deadwood as narrow buffers due to blowdowns but were, overall, more resistant to wind-felling. It is clear from our study, that wider buffers are currently a safer strategy for riparian management that aims to sustain provision of deadwood and other ecological objectives continuously on the long term.

**Supplementary Information:**

The online version contains supplementary material available at 10.1007/s13280-022-01793-1.

## Introduction

In Sweden, forestry operations are carried out on hundreds of thousands of hectares of forestland annually (Skogsstyrelsen 2020). Due to the high density of streams in boreal forest (Lidberg et al. 2020) the large-scale forestry operations affect many kilometers of waterways every year. It is generally accepted that leaving strips of unharvested riparian forest, called “riparian buffers”, along streams and rivers will mitigate or prevent the negative effects of forestry on water quality and quantity, and aquatic and riparian ecology (Lee et al. 2004; Ring et al. 2017; Kampf et al. 2021). However, recent research has shown that there is a large variation in the efficacy of buffers in protecting aquatic and riparian ecosystems in managed forested landscapes. This variation is caused by buffer width and tree species composition, local riparian properties (e.g., slope, soil moisture) as well as catchment-scale variables (Oldén et al. 2019a, b; Jyväsjärvi et al. 2020; Chellaiah and Kuglerová 2021). In addition, Hasselquist et al. (2021) showed that in Sweden, many riparian forests along small streams are still influenced by a legacy of past management that performed commercial forestry operations (thinning and cleaning, and potentially planting of commercially important conifers) all the way to the edge of streams. This has resulted in supressing natural disturbances and dynamics of riparian forests that would, without management, have more canopy gaps, larger variation in tree dimensions as well as higher volumes and larger heterogeneity of deadwood (Lundqvist 2022). Instead, mature riparian forests in production stands typically have single-story canopies and spruce dominance, as well as reduced input of deadwood in riparian forests. Thus, buffers created today, and for the next few decades, carry the legacy of past management and this, in turn, will have consequences for their functionality (Kuglerová et al. 2021).

The most recent updates on Swedish buffer guidelines are presented as the Strategic Management Objectives (SMOs) developed by the forest sector (Andersson et al. 2013). Compared to many countries that prescribe riparian buffer width (Lee et al. 2004; Ring et al. 2017; Kampf et al. 2021), instead Sweden prescribes ecological functions to be maintained. Buffers should (1) provide shade, (2) prevent sediment transport, (3) protect biodiversity, (4) maintain important biogeochemical cycling, (5) provide food for aquatic organisms and (6) provide deadwood. Deadwood is a particularly important component that should be sustained by riparian buffers in Swedish conditions because production forestry practices has significantly reduced deadwood recruitment to the forest floor and to streams (Siitonen et al. 2000; Dahlström et al. 2005; Gustafsson et al. 2020). However, deadwood is a vital element of stream and riparian ecosystems, being an important substrate for many species, and supplying slowly decomposing organic matter (Gurnell et al. 1995; Hylander et al. 2005; Johnson and Almlöf 2016). Further, in-channel wood is an essential component of stream geomorphology through its effect on pool-riffle formation and lateral channel movement (Motgomery et al. 1995; Martens et al. 2020), morphological aspects that play a vital role in providing habitat for many taxa as well as affecting biogeochemistry of headwater streams (Bisson et al. 1984; Bilby and Ward 1991). A large number of species are threatened in Sweden due to the lack of deadwood, and this includes many riparian and aquatic organisms (Eide et al. 2020; Gustafsson et al. 2020). In an attempt to sustain long-term and continuous deadwood recruitment, as well as preserve other environmental values, retention patches are often included as part of the sustainable forestry model in Sweden. This assumes that wood will be continuously provided in retention patches due to natural tree mortality, snow/ice and insect damages, wind-felling, and in the case or riparian zones, bank collapse and soil movement due to increased wetness (Bisson et al. 1984). In reality, deadwood is typically recruited soon after final felling, when trees in retention patches, including riparian buffers, blow down (Dynesius and Hylander 2007; Mäenpää et al. 2020; Hasselquist et al. 2021). The question remains, whether this strategy provides ecologically sufficient quantity, quality, and timing of deadwood recruitment, especially over the long term.

Some evidence suggests that the volume of wind-felled wood provided by riparian buffers is determined by their width (Mäenpää et al. 2020; Chellaiah and Kuglerová 2021). The assumption is that the wider the buffer is, the less blowdown it should experience and, thus, less large deadwood would be recruited by wind-felling. However, this phenomena was only documented in buffers 30 m wide on each side of the stream (Bahuguna et al. 2010; Mäenpää et al. 2020; Peura et al. 2020), and such wide buffers are very rare in Sweden along small streams (Kuglerová et al. 2020). Contrastingly, the narrower the buffer is, the more windthrows likely occur, but a narrow buffer also contains less trees that could potentially become deadwood in the future (Jyväsjärvi et al. 2020; Chellaiah and Kuglerová 2021). In Sweden, we currently do not know how the width of riparian buffers affects the amount of deadwood provided to the stream and riparian zone due to wind-felling. This is vital to know in the light of a recent study, which showed that average buffer width along small streams in Sweden is just 4 m on each side of the stream (Kuglerová et al. 2020). Such narrow buffers are likely at high risk for severe windthrows shortly after their establishment, leaving very few or no standing trees after a storm. Although this would be beneficial for the deadwood provision in the short term, it would compromise deadwood diversity (in terms of tree species and decomposition status or age) and hence quality for organisms, and the potential for future provision of deadwood due to agents other than wind-felling. In addition, high volume of windthrows would negatively affect other riparian buffer functions, including shading, sediment transport control, and provision of leaf litter.

Several other factors besides width are necessary to take into account when designing buffers. For example, researchers have been suggesting leaving wider buffers on riparian areas with steep slopes or very wet soils compared to flatter and dryer areas (Kuglerová et al. 2017). Steep stream banks can be a problem because, if harvested, they can be a large source of fine sediments (Lee et al. 2004). Furthermore, wet riparian zones are more susceptible to soil rutting (Ågren et al. 2015) and exert a stronger control over water chemistry and biodiversity than drier riparian zones (Kuglerová et al. 2014). What we do not know is whether topographic and soil conditions determine the susceptibility of riparian buffers to windthrows, and therefore if these factors can be also related to short-term deadwood recruitment. In strictly terrestrial ecosystems, predicting windthrows from landscape characteristics seems to be difficult, due to the interplay of many factors, including wind intensity, exposure, and stand or individual tree properties (Ruel 2000; Zeng et al. 2004; Bouchard et al. 2009). Yet, some parameters have been identified as increasing the risk for blowdown, including size and slope of clearcuts, or tree species (Everham and Brokaw 1996; Ruel et al. 2001; Mäenpää et al. 2020). The latter is especially true for large individuals of Norway spruce (Zeng et al. 2004), a tree which is dominant in riparian forests in Sweden (Hasselquist et al. 2021).

In this paper, we aim to describe the deadwood recruitment in recently created riparian buffers along small streams in Sweden. We have three main questions: (1) How much deadwood is provided by newly established riparian buffers? (2) Is the provision of deadwood related to the buffer width? and (3) Are there landscape characteristics that are correlated with the volume of deadwood in riparian buffers? We answer these questions by using two data sets, one investigating 28 recently established riparian buffers along small streams in northern Sweden (*regional data*). The second data set comes from a riparian buffer experiment in the vicinity of the Krycklan Catchment Study, the *Trollberget Experimental Area* (Laudon et al. 2021). At Trollberget, six reaches of the same stream received either a 5- or 15-m-wide riparian buffer on each side of the stream (3 replicates of each width in an alternating fashion), and were inventoried for deadwood before and after the adjacent stand was harvested. We hypothesize that riparian buffers will provide relatively large volumes of deadwood within the short time frame due to blowdown of large exposed spruce. Furthermore, we predict a bell-shaped relationship between buffer width and volume of deadwood, with medium-width buffers having higher recruitment of deadwood than both narrow and wide buffers because narrow buffers have fewer trees to blow down and wide buffers will be more resistant to windthrows. Finally, we hypothesize that slope and soil wetness of the riparian area, as well as clearcut size and time since establishment will be positively correlated with the volume of deadwood in buffers due to inherently lower tree stability on steep slopes and in wet soils, higher wind exposure in larger clearcuts, and longer wind exposure in older buffers.

## Materials and methods

### Regional data

**Fig. 1:**
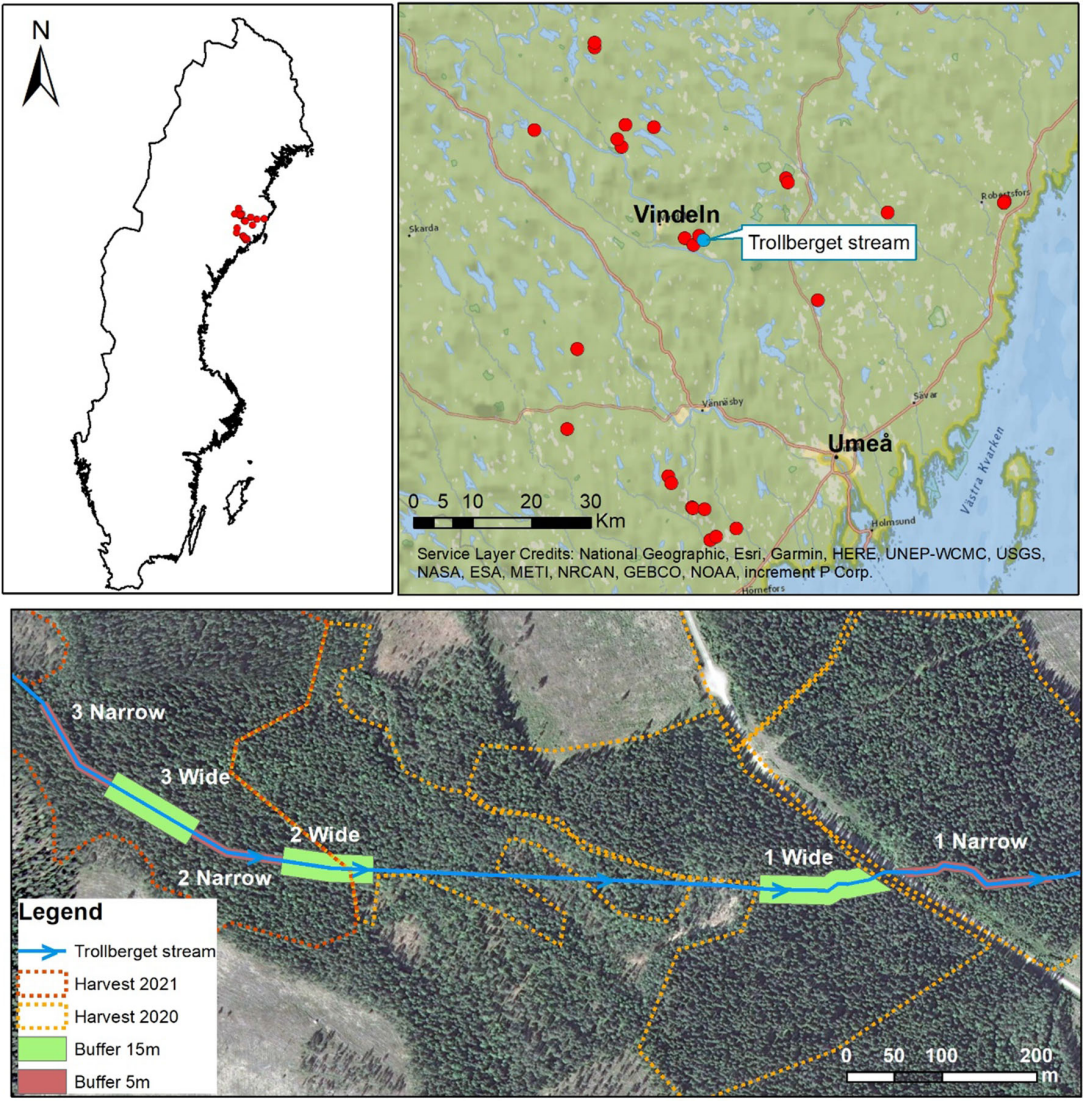
Location of the study sites in Sweden (upper left). Regional-scale data were collected at 28 sites (red dots) along headwater streams in Västerbotten County. The Trollberget experimental stream (blue dot) included six reaches that received either narrow (5 m) or wide (15 m) riparian buffers when the adjacent forest was harvested (bottom). The boundary of the clearcut harvest in 2020 (orange) and 2021 (red) is displayed over an aerial photo taken before the harvest

This data were collected during September 2020 at 28 sites located in Västerbotten County, Sweden (Fig. 1). The streams selected for this study have been used in previous work (Kuglerová et al. 2020; Chellaiah and Kuglerová 2021) and represent headwater streams. All of the sites were situated in clearcuts harvested between 2010 and 2020 (Fig. S1), mostly on land owned by forest companies. Pre-harvest data on the stands were not available, but after the harvests, riparian buffers of various widths were left along the streams, with the riparian forest structure dominated by single-storied, mature Norway spruce.

At each site, a 50-m-long longitudinal stream reach was established for the riparian buffer width and deadwood surveys. We measured *current buffer* width at the beginning (0 m), middle (25 m), and end (50 m) of the reach on both sides of the stream; the outer edge of the buffer designated by the outermost standing trees. We also estimated the *original buffer* width at the same locations (0, 25, and 50 m); designated by the outermost uprooted or broken trees (if present). The diameter at breast height (DBH) was measured on all deadwood both in water and on land, which was rooted within the original buffer. The length, tree species, and type of damage (uprooted, stem breakage or cut) was registered for all deadwood with a DBH > 5 cm or length > 1 m; the position of the deadwood was also measured, as fully laying on the ground, suspended above the channel or ground, or standing upright. The stage of decomposition was assessed by using Maser et al. (1979) decay classification system for logs (class 1–5) and snags (class 6). The volume of the deadwood (m^3^) was calculated by using functions from Näslund (1947) for spruce, birch and pine, from Eriksson (1973) for aspen and alder, and cylinder function for deadwood that was not possible to identify to species. The volume of deadwood was then expressed per hectare (m^3^/ha), to account for differences in buffer width, and thus different areas in which deadwood was recorded.

We derived several topographic and landscape variables for each of the 28 sites from spatial data. First, we obtained the year of the harvest and the size of the clearcut at each site from data downloaded from the Swedish Forest Agency database (Skogsstyrelsen 2021). Second, we delineated an area of 20 m lateral distance on each side of the stream along our 50 m reach (0.2 ha) in which we calculated the maximum slope of the riparian area (the steepest pixel within the riparian zone) and average soil wetness. The slope was derived from digital elevation model (DEM) with 2 × 2 m resolution and the wetness was obtained from the Swedish Wetness Map, that is derived by machine learning algorithms based on 2 × 2 DEM and other landscape, topographic and hydrological variables (Lidberg et al. 2020).

### Trollberget experimental stream

The second deadwood data set comes from the Trollberget Experimental Area (Laudon et al. 2021) where riparian buffers were created when the adjacent forest was clearcut in July 2020 and February 2021 (Fig. 1). The Trollberget stream is a headwater stream (0.63 km^2^ catchment area) situated in the same region where we collected the regional data (Fig. 1). Six adjacent 100-m-long stream reaches received either a 5-m (narrow) or 15-m (wide)-wide buffers on each side of the stream. We inventoried all in-channel deadwood in those six reaches in June 2020 before the harvest was performed and the buffers were created. In November 2020, we re-inventoried the two most downstream reaches (1 Wide and 1 Narrow, Fig. 1) after the harvest of adjacent stand was completed in July 2020. In September 2021, we re-inventoried the four upstream reaches (Fig. 1) after the harvest in February 2021. During all field surveys (before and after harvest) we recorded the diameter, length, species and type (coniferous vs. deciduous), and position (within bankfull stage channel, bridge across the channel, or in the water) of all in-channel deadwood pieces that had a DBH > 5 cm or length > 1 m. We did not record deadwood that was located in the riparian area and did not reach the channel. Consequently, we calculated volume (m^3^) and number of deadwood objects at each stream reach using the same formulas as above. We did not correct for the surveyed area because we only recorded deadwood objects that were in or above the channels and this linear distance was the same across all six stream reaches (100 m). It is important to note that soon after the buffers were created in July 2020 at the two most downstream reaches, two severe storms occurred in the area in October and November, with high wind speeds and a record high stream flow (Laudon et al. 2021). The deadwood that was created along those two reaches by the storms was consequently salvage logged by the landowner after our inventories were finished.

### Statistical analyses

#### Regional data

At each of the 28 sites, we calculated the average *current* and average *original* buffer width from the six measurements (3 marks and 2 sides of the stream), and buffer width loss (due to blowdown) as the difference between the original and current width. To test if narrower buffers are more susceptible to wind damages, we related the buffer width loss to the original buffer width by creating an ordinary least square regression model (OLS). For this OLS model, we only used a subset of 16 sites, which had some buffer width loss recorded, to avoid zero-inflated data. Buffer width loss was log-transformed before the OLS to accommodate normal distribution of residuals. We further tested whether the volume of deadwood per hectare was related to the original buffer width. For this analysis we only used the deadwood classes 1 and 2 (henceforth called “fresh deadwood,” Peura et al. 2020), to be sure that these deadwood objects were wind-felled after the buffer was created. We assumed that deadwood from classes 3–5 were recruited to the streams and forest floor earlier during the stand development (Maser et al. 1979). Log-transformed volume of fresh deadwood per ha (m^3^/ha) was used as the dependent variable and original buffer width as the independent variable in an OLS model.

To test whether site characteristics also affect the deadwood volumes we performed partial correlation analyses between the volume of deadwood/ha and four topographic/landscape variables: year of the harvest, size of the clearcut, maximum riparian slope and average riparian soil wetness. We used partial correlations to control for the effects of buffer width on deadwood volume and used Spearman correlation tests because most of the data were not normally distributed. All statistical analyses were done in RStudio (R Developmental Core Team 2019) and the partial correlations were performed using the package *ppcor* (Seongho 2015).

#### Trollberget experimental stream

We used analyses of variance (Anova) to test the effect of buffer width (15 vs. 5 m) and harvest period (before vs. after) for the Trollberget data. We used total volume of deadwood (m^3^) as well as number of recorded deadwood objects as response variables, and buffer width and harvest period (and their interaction) as explanatory variables in two separate Anova. Volume of deadwood was log-transformed to meet the assumption of normality of residuals. We further calculated number of objects of deciduous vs. coniferous deadwood, as well as number of objects located within the bankfull channel, in water or forming a bridge, per stream reach and per harvest period. We then expressed the changes in those deadwood metrics (species and position) as a proportion of change between the two harvest periods per buffer width. Finally, we calculated descriptive statistics (average ± SD) for the length and diameter for all the deadwood objects for each stream reach and period.

## Results

### Regional data

**Fig. 2:**
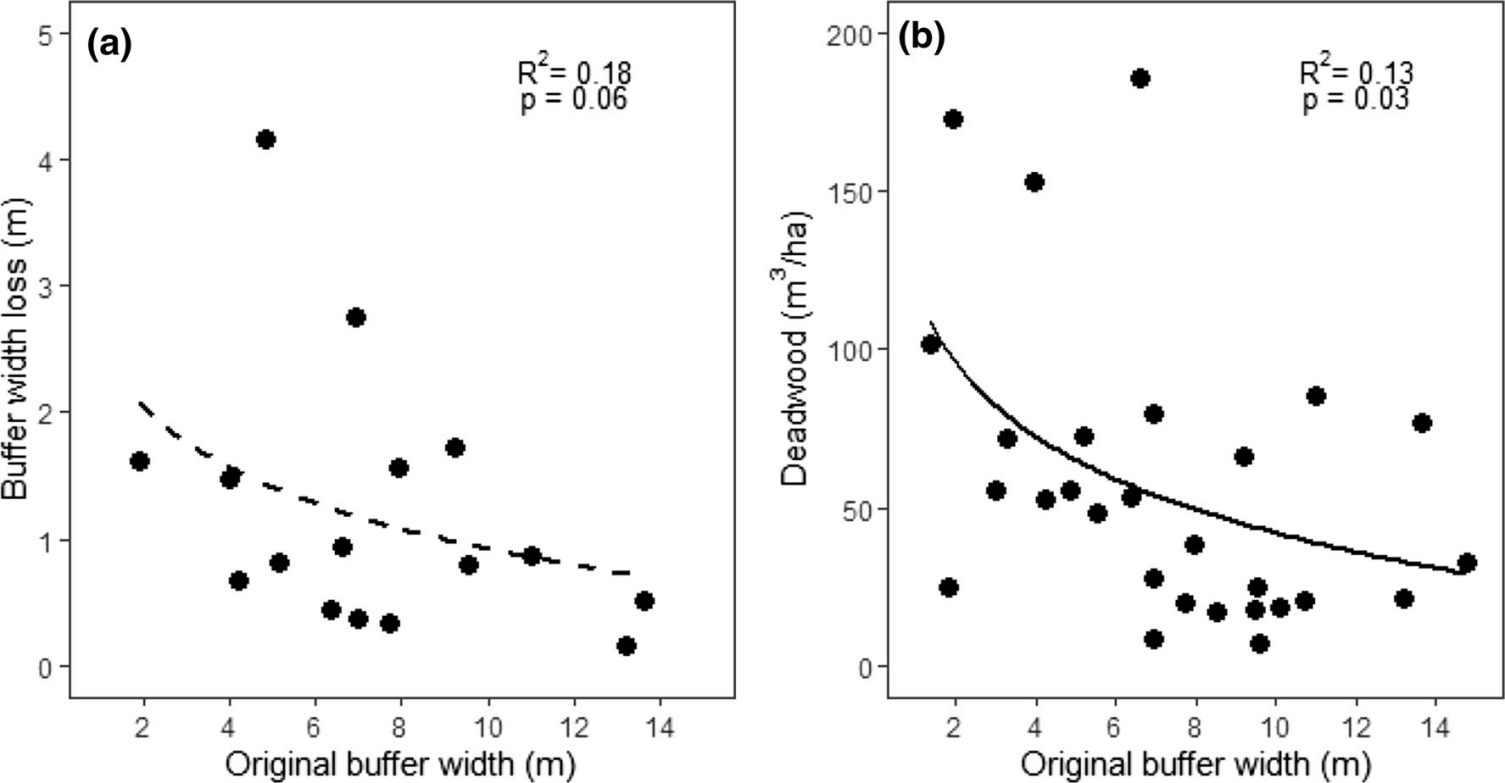
The relationship between the original width of the riparian buffers and (**a**) the buffer width loss calculated as the difference between original and current buffer width, and (**b**) the volume of fresh deadwood per hectare (m^3^/ha) recorded in the riparian buffers. Both relationships were tested with ordinary least square regression with regression parameters presented in the figures. Full and dashed lines represent significant (*p* < 0.05), and non-significant regression lines, respectively

Across all 28 sites, the average (± 1 SD) original and current buffer widths were 7.3 (± 3.6) m and 6.6 (± 3.9) m, respectively. On average (± 1 SD) the loss of the buffer width was 0.7 (± 1) m, including 12 sites that experienced no buffer width loss. After removing those 12 sites, the average buffer width loss was 1.2 (± 1.1) m. The highest width loss recorded was 4.2 m. Buffer width loss was negatively related to the original buffer width; the wider the original buffer, the lower the buffer width loss (OLS: *t* = − 2.05, *p* = 0.06), although the variation in the data was large especially with decreasing original buffer width (Fig. 2a). Original buffer width was also negatively related to the volume of fresh (class 1–2) deadwood per hectare (OLS: *t* = − 2.26, *p* = 0.03, Fig. 2b).

Across the 28 sites, we recorded 570 deadwood objects with average length (± SD) of 12.0 (± 6.1) m and average DBH of 16.9 (± 8.9) cm. Sixty-two percent of all deadwood objects were Norway spruce, 30% were downy birch and the remaining 8% were a combination of grey alder, Scots pine, trembling aspen and rowan (Table 1). Seventeen of the deadwood objects (3%) were not possible to identify to species because they were too decomposed. Over 70% of all deadwood objects belonged to the decomposition class 1–2, and classified as fresh deadwood (wind-felled after the buffer was created). An additional 16% belonged to the class 3 and only 7.7% of the recorded deadwood was classified as old deadwood, belonging to classes 4 and 5 (Table 1). Five percent of the deadwood were snags (class 6). Most deadwood was laying on the ground (88%) and was uprooted (56%, Table 1).

**Table 1:** Characteristics of all deadwood objects found in riparian buffers across the 28 study sites in the regional data. The total numbers and percentages of totals are presented for the different tree species, decomposition classes, positions and damage causes separately. Average (± SD) length (m) and DBH (cm) for each species are also presented

	Deadwood objects (#)	% of totals	Length (m) mean ± SD	DBH (cm) mean ± SD
*Species*				
Total	570	100	12.0 ± 6.1	16.9 ± 8.9
Norway spruce (*Picea abies*)	355	62.3	12.9 ± 6.3	18.5 ± 9.8
Downy birch (*Betula pubescens*)	170	29.8	11.1 ± 5.7	13.7 ± 6.3
Grey alder (*Alnus incana*)	17	3.0	9.7 ± 3.4	13.8 ± 5.1
Scots pine (*Pinus sylvestris*)	9	1.6	13.6 ± 4.9	19.3 ± 3.4
Trembling aspen (*Populus tremuloides*)	1	0.2	12	10.6
Rowan (*Sorbus aucuparia*)	1	0.2	5	8.2
Unknown	17	3.0	4.6 ± 2.5	18.1 ± 9.8
*Decomposition class*				
1 (bark intact, twigs present, texture intact)	99	17.4		
2 (bark intact, twigs absent, texture intact to soft)	306	53.7		
3 (bark trace, twigs absent, texture large pieces)	92	16.1		
4 (bark absent, twigs absent, texture soft)	31	5.4		
5 (bark absent, twigs absent, texture powdery)	13	2.3		
6 (snags)	29	5.1		
*Position*				
Laying on the ground	502	88.1		
Standing (rooted)	35	6.1		
Suspended (uprooted but supported)	33	5.8		
*Damage*				
Cut	16	2.8		
Stem broken	227	39.8		
Uprooted	317	55.6		
Unknown	10	1.8		

After controlling for the effect of the riparian buffer width using partial correlations, the strongest relationship was between deadwood volume and maximum riparian slope (*r* = 0.36, *p* = 0.07), suggesting that with increasing slope of the riparian area, the volume of deadwood increases. The second strongest relationship was for riparian soil wetness (*r* = − 0.22, *p* = 0.28) but this correlation was weak. Finally, year of harvest (*r* = 0.11, *p* = 0.55) and size of the clearcut (*r* = − 0.01, *p* = 0.97) had very weak correlations with the volume of deadwood.

### Trollberget experimental stream

**Fig. 3:**
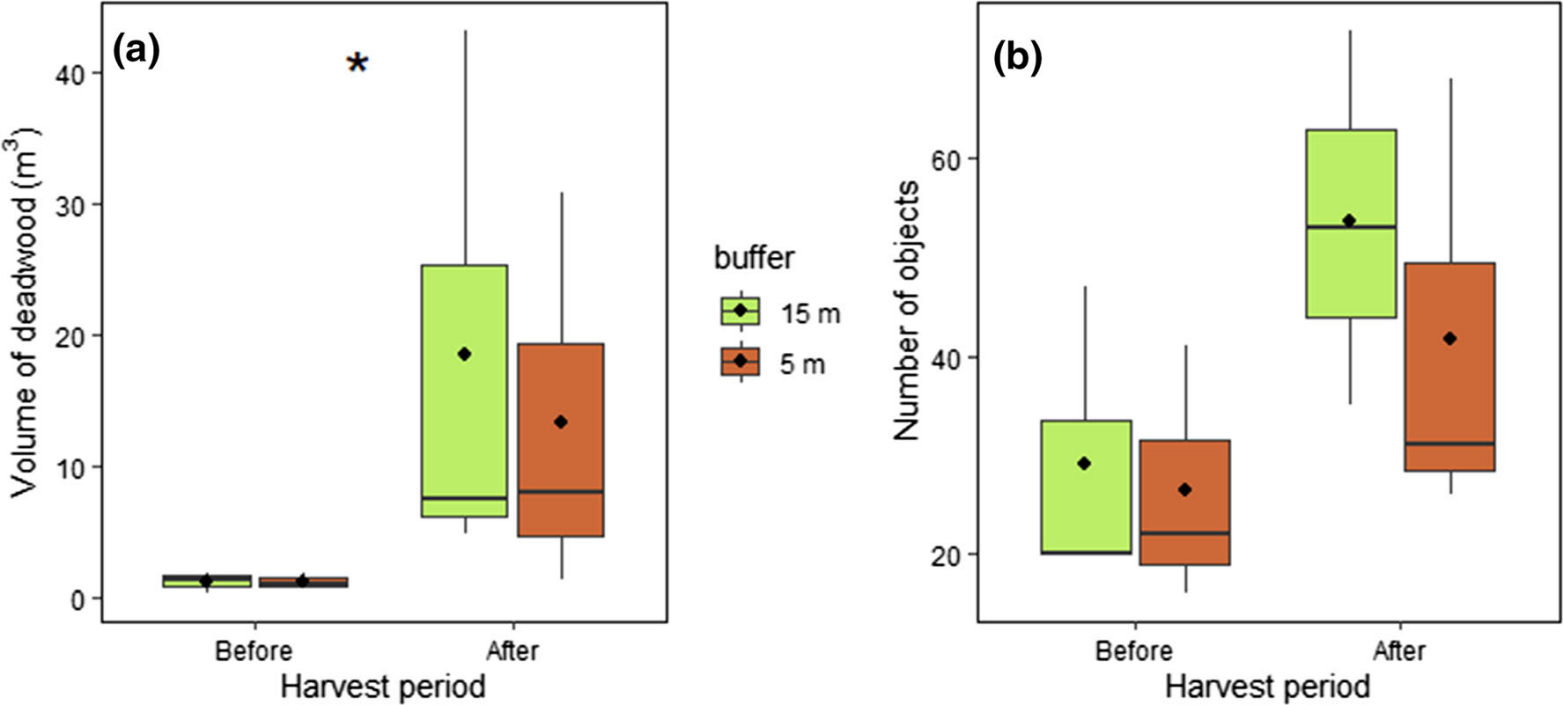
Volume in m^3^ (**a**) and number of objects (**b**) of deadwood recorded per 100 m stream reach in the six reaches at the Trollberget Experimental Area. Three reaches received a narrow (5 m) and three reaches received a wide (15 m) buffer when the adjacent forest was harvested. Deadwood was measured and calculated for before and after harvest periods. Horizontal lines represent medians, and black points represent averages for buffer type and treatment with quantiles indicated by the boxes. Error bars represent minimums and maximums. *indicates significant effect of harvest period (*p* < 0.05)

All six stream reaches had relatively low volumes of in-channel deadwood before the harvest and establishment of the buffers, ranging between 0.2 and 1.8 m^3^ and between 16 and 47 deadwood objects per 100 m reach (Table 2). Deadwood volume and number of objects increased substantially after the harvest to 1.2–43.3 m^3^ (Anova: *F* = 13.6, *p* = 0.004, Fig. 3a), and to 26–73 objects per 100 m reach (Anova: *F* = 4.2, *p* = 0.07, Fig. 3b, Table 2). This increase was mostly caused by an enormous increase at the two most downstream reaches (1 Narrow and 1 Wide, Fig. 1) that were already harvested before the storms in the autumn of 2020. In particular, the deadwood volume in reach “1 Wide” increased from 0.2 before to 43.3 m^3^ after the harvest (from 20 to 73 pieces of deadwood), which corresponds to a 181 fold increase in deadwood volume (Table 2). In “1 Narrow,” the deadwood volume increased from 1.0 m^3^ before to 30.8 m^3^ after the harvest (from 41 to 68 pieces of deadwood), which corresponds to a 31 fold increase (Table 2). All four upstream reaches that had intact forest during the storms in 2020 but were harvested afterwards also experienced increases in the deadwood volumes and number of deadwood objects after harvest, but not as high as at the two downstream reaches (Table 2). Buffer width did not strongly affect the volume of deadwood (Anova: *F* = 0.06, *p* = 0.82, Fig. 3a) or the number of objects (Anova: *F* = 0.5, *p* = 0.48, Fig. 3b) that were recorded before and after the harvest. The deadwood also increased in size (length and diameter) in all reaches after harvest (Table 2).

**Table 2:** Total volume (m^3^), total number of objects, mean (± SD) length (m) and mean (± SD) DBH (cm) of deadwood recorded at the six reaches on the Trollberget experimental stream. The numbers are presented for each 100 m reach before and after harvest of the adjacent stand. The reaches with † were already harvested when extreme storms occurred in the autumn of 2020

Reach	Harvest period	Buffer width (m)	Total volume (m^3^)	Total objects (#)	Length (m) mean ± SD	DBH (cm) mean ± SD
1 Narrow	Before	5	1.0	41	3.7 ± 4.3	9.3 ± 3.8
	After^†^	5	30.8	68	15.2 ± 10.7	20.4 ± 11.1
2 Narrow	Before	5	1.8	16	8.3 ± 7.3	15.3 ± 6.4
	After	5	8.0	31	12.2 ± 7.7	19.3 ± 8.5
3 Narrow	Before	5	0.7	22	4.2 ± 4.3	10.7 ± 4.3
	After	5	1.2	26	5.0 ± 4.3	10.6 ± 6.0
1 Wide	Before	15	0.2	20	2.9 ± 3.2	7.7 ± 2.5
	After^†^	15	43.3	73	17.5 ± 10.3	24.2 ± 10.9
2 Wide	Before	15	1.8	20	7.7 ± 8.3	10.6 ± 6.9
	After	15	7.5	35	11.3 ± 8.1	14.5 ± 9.1
3 Wide	Before	15	1.3	47	4.0 ± 4.5	10.5 ± 4.0
	After	15	4.8	53	5.9 ± 6.4	13.1 ± 6.0

**Fig. 4:**
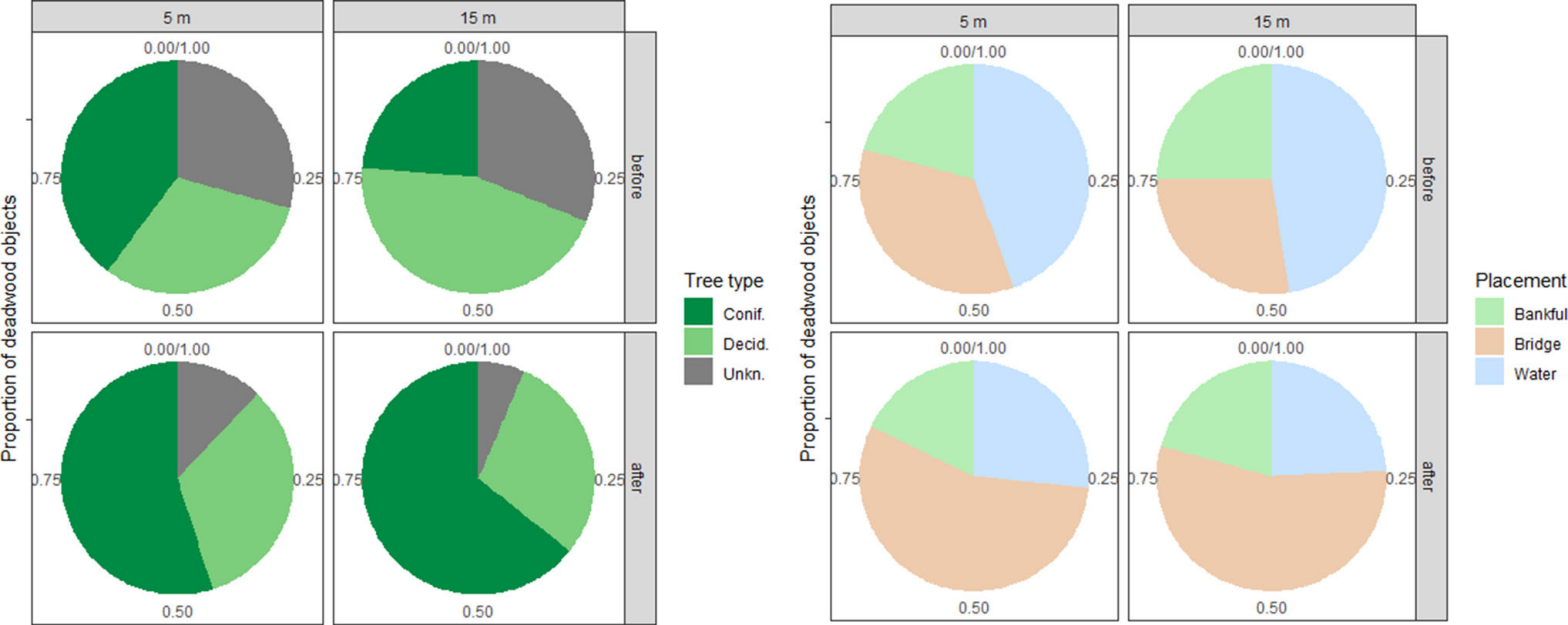
Average proportions of deadwood objects found at the 5 and 15 m buffer reaches at Trollberget experimental stream divided by type: coniferous, deciduous and unknown wood (green shades to the left). Average proportions of deadwood objects per buffer width divided by position in respect to the stream channel (bankfull, bridge and in water) before and after harvest (pastel shades to the right)

Before the harvest, the majority of the deadwood objects were classified as deciduous and unknown wood, and were located in water (Tables S1, S2). Deciduous species were dominantly represented by downy birch (Table S1). After harvest, the majority of the deadwood was coniferous (mainly Norway spruce, Table S1) and formed bridges (Table S2). The increase in the proportion of coniferous deadwood from before to after harvest was larger for the reaches with 15 m wide buffers where the coniferous deadwood increased by 40% on average, while at the reaches with 5 m wide buffers this increase was 15% (Fig. 4). Similarly, the increase in deadwood that formed bridges was larger for the 15 m buffers reaches (27% increase on average), compared to 5 m buffers (21% increase on average, Fig. 4).

## Discussion

Our study clearly shows that recently established riparian buffers are able to provide deadwood—at least in the short term, one of the core Strategic Management Objectives (SMOs) for riparian buffer practices in Sweden (Andersson et al. 2013). We recorded large volumes of deadwood in buffers in both studies—at the regional scale as well as at the Trollberget experimental stream. The Trollberget experiment further showed very low volumes (< 1.8 m^3^ per 100-m-long reach) of deadwood in streams before harvest, presumably caused by the historical suppression of deadwood recruitment by Swedish forest management in production stands (Siitonen et al. 2000; Dahlström et al. 2005; Gustafsson et al. 2020). After the buffers were created at Trollberget, windthrows caused up to 181 times more deadwood volume to be recruited compared to pre-harvest conditions (up to 43 m^3^ per 100-m-long reach). The regional-scale data also point towards low volumes of deadwood before harvest, as the volume of old deadwood (decomposition class 3–5) in the buffers was on average 12.7 m^3^/ha, which is 4.5 times lower than the volume of fresh deadwood (57.3 m^3^/ha on average for class 1–2). It is difficult to judge whether the quantities of the deadwood found in our study are ecologically sufficient because we did not evaluate its effects on habitat and organisms. Nevertheless, comparing to quantities reported in streams situated in pristine boreal forests (Liljaniemi et al. 2002; Dahlström and Nilsson 2004) it seems that our streams with recently established riparian buffers and with high volumes of wind-felled trees are on the way to recovery from intensive management in riparian zones (Hasselquist et al. 2021) towards volumes of deadwood typical of old growth forests.

**Fig. 5:**
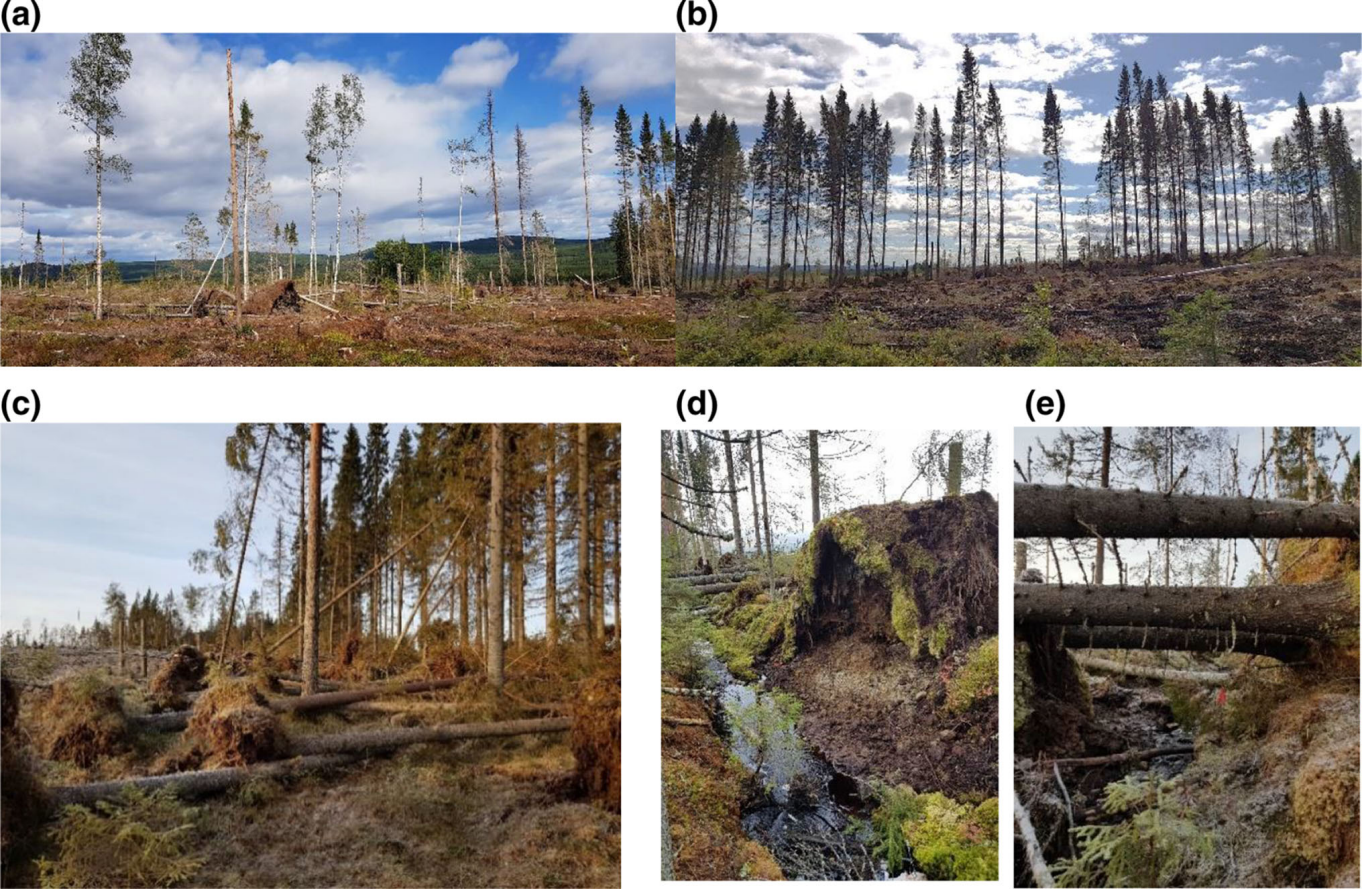
5-m (**a**) and 15-m (**b**)-wide riparian buffers established at the Trollberget experimental stream at the two most downstream reaches (1 Narrow, 1 Wide) photographed after the two storms in October and November 2020. Only several trees remain standing in the 5 m buffer, leaving no continuous buffer along this stream reach (**a**), while a ca. 10 m wide, but patchy buffer, remained at the 15 m buffer reach (**b**). Most deadwood was recruited after the storms causing large root wads and sediments to be exposed, either at the outer buffer edge along the 15-m-wide buffer (**c**) or by the stream edge at the 5-m-wide buffer (**d**). Many blown-down trees were left suspended above the stream channels (**e**). Note that most of the deadwood in the 2 reaches affected by storm damage was salvage logged by the land owner (Photographs **a** and **b** were taken after salvage logging while **c**, **d** and **e** before). Photos by L. Kuglerová

While increasing volume of deadwood in streams and riparian areas is certainly positive news and could almost be viewed as a passive restoration measure, such high volume of windthrows as observed here can lead to environmental problems. First, if most of the trees that were left in the buffer blow down, other riparian functions required from buffers are compromised. For example, shading and water temperature regulation are better maintained by wide and intact buffers compared to narrow or partially harvested ones (Oldén et al. 2019b; Jyväsjärvi et al. 2020; Chellaiah and Kuglerová 2021). We showed that the narrower the buffers were to begin with, the more susceptible they were to wind-felling, and this will inevitably lead to a decreased ability to provide shade. Further, blown-down trees were associated with uprooting (Fig. 5c, d), and at the stream edges this can disturb the channel itself leading to large pulses of sediments directly to the streams (Grizzel and Wolff 1998; Hasselquist et al. 2021). In the Trollberget experiment, we recorded eight such damaging root wads along the stream edge in the 5 m buffers (Fig. 5d), while only three root wads at the stream edge were observed across the reaches with 15 m buffer (data not shown). Prevention of sediment loading and maintenance of shading are the primary reasons for protecting riparian forests (Lee et al. 2004; Ring et al. 2017), and are listed, together with provision of deadwood, in the SMOs for water protection in Sweden (Andersson et al. 2013). From the results presented here, it seems that those SMOs—provision of deadwood and prevention of sediment transport/maintenance of shade—are somewhat mutually exclusive in contemporary buffer practices in Sweden. Finally, the risks associated with large volumes of blown-down trees are also economic. Landowners in Sweden are obligated to remove large volumes (> 5 m^3^/ha) of fresh coniferous deadwood in order to prevent bark beetle infestation. We saw salvage logging at the Trollberget experimental stream, and this additional operation is associated with an extra cost as well as an additional disturbance since heavy harvest machines enter the site again (Hasselquist et al. 2021). Clearly riparian buffers need to be wider to begin with, to allow for provision of deadwood while not compromising their other functions.

The widest buffers we were able to evaluate in this study were 15 m wide. At Trollberget, those buffers experienced similar volume of blown-down trees as the 5-m-wide buffers, but most of them occurred on the outer buffer edges (Fig. 5c), resulting in about 10-m-wide buffer (Fig. 5b) with only minor disturbances to the stream channel. It is important to note that at the Trollberget site, we only measured wood that reached or crossed the stream channel. Although this was the majority of blowdown trees at the site, some trees fell in the opposite direction. Therefore, the total wind-felling in the riparian buffers was likely higher and the difference between 5- and 15-m-wide buffers could be larger. We are not able to assess the riparian deadwood due to the salvage logging after our measurements. The regional-scale data also showed higher resistance of wider buffers against wind-felling, since wider buffers had lower volume of fresh deadwood in general and lower buffer width loss, indicating that wind-felling in wider buffers occurs also within the buffer (not only on the edges). The gaps provided by those windthrows should experience recruitment of understory deciduous vegetation (Mallik et al. 2014). This should, on the long term, support a more heterogeneous riparian forest, provide more varied deadwood throughout the rotation, and presumably create a better basis for a functional buffer zone after the adjacent forest is harvested again in another 80–100 years if the sites continue to be in an even-aged, rotation forestry system (Hasselquist et al. 2021). This aspect is essential to include in best-practice-management considering that vast majority of the deadwood we encountered in both studies was fresh spruce and most of the new deadwood was suspended above the streams (Fig. 5e), not currently benefiting aquatic habitat and organisms (Grizzel and Wolff 1998; Rossetti de Paula et al. 2020). Ideally, deadwood should vary in age, species composition and location within the riparian-aquatic environment to support high diversity of organisms utilizing the wood in the decades to come (e.g., Bisson et al. 1984; Dynesius and Hylander 2007). Unfortunately, buffers wider than 15 m are rare in the region along headwater streams, and much narrower buffers are the norm (Kuglerová et al. 2020). This was also seen in our regional-scale study where the average original buffer width was 7.3 m and the widest buffer we found was just under 15 m. Therefore, we were not able to evaluate the deadwood provision in as extensive a range of buffer widths as in other studies (Grizzel and Wolff 1998; Jyväsjärvi et al. 2020; Mäenpää et al. 2020), and are not able to provide insights about the width of the buffers that could prevent short-term windthrows completely. The limited range of the buffer widths we found might also be the reason that we have not seen the predicted bell-shaped relationship between buffer width and deadwood volume that was observed for buffers up to 30 m wide (Mäenpää et al. 2020). Buffers that are 30 m or wider are recommended by many studies from the boreal region to preserve all ecosystem functions and protect biodiversity of streams and riparian zones (Selonen and Kotiaho 2013; Oldén et al. 2019b; Jyväsjärvi et al. 2020). While 15 m buffers might seem to be a reasonable solution for the provision of deadwood based on the results of this study, they are likely not able to sufficiently sustain all ecosystem functions, especially protecting biodiversity (Selonen and Kotiaho 2013).

We further expected that larger clearcuts might be associated with more windthrows (Elie and Ruel 2005; Mäenpää et al. 2020). The range of the clearcuts we inventoried in the regional-scale dataset was relatively wide (1.9–72.6 ha) but with no strong effect on deadwood volume. Ruel et al. (2001) described that topographic variables such as steepness, hill form and orientation of the clearcut are the factors that most influence wind speed, which can explain why size on its own did not correlate well with deadwood volumes in our study. The year of harvest also had no relationship with deadwood volumes, even though we expected that older buffers would have more wind-felling due to longer time for wind exposure. Nevertheless, looking at the extensive wind-felling that occurred at the Trollberget sites just three months after it was harvested, it seems that one event can cause most of the wind-damage. Most likely, our sites in the regional dataset, that were harvested in the past decade, probably experienced all initial windthrows already (Jönsson et al. 2007; Bahuguna et al. 2010). Whether those sites will experience further deadwood recruitment over the long term due to other agents than wind-felling (e.g., natural tree mortality, snow and insect damages, bank collapse) remains to be seen. There were indications that both riparian slope and soil moisture have some correlation with deadwood recruitment. The negative correlation between deadwood volume and average soil moisture in the riparian areas was surprizing, as we expected wetter soils to have less root stability and easier uprooting (Everham and Brokaw 1996). Our results might reflect the effect of too wet riparian soils that can actually hinder tree growth (Tiwari et al. 2016), and thus there are fewer and smaller trees available to become deadwood. Steep slopes are more susceptible to wind-felling compared to flat ones (Everham and Brokaw 1996) and we have some support that the steepest riparian slopes promoted blown-downs. Nevertheless, all topographic factors interact to determine susceptibility to windthrow (Ruel 2000; Zeng et al. 2004; Bouchard et al. 2009), and this is probably why we found rather weak correlations between the tested variables and deadwood. One aspect that we did not evaluate in our study was the effect of initial riparian forest conditions on the deadwood recruitment. Stand conditions largely influence the severity of wind damages, including the effects of tree species and density, stand mixture and age (Everham and Brokaw 1996; Zeng et al. 2004). Unfortunately, we had no access to such data for the riparian stands before they became a buffer. Our sites were (a) all dominated by mature spruce, (b) owned by large forest companies, (c) of similar age (mature for harvest in northern Sweden), and (d) with similar historical legacy (underwent thinning, cleaning). We thus believe that we have minimized the variation in stand conditions.

## Conclusions and management implications

**Fig. 6:**
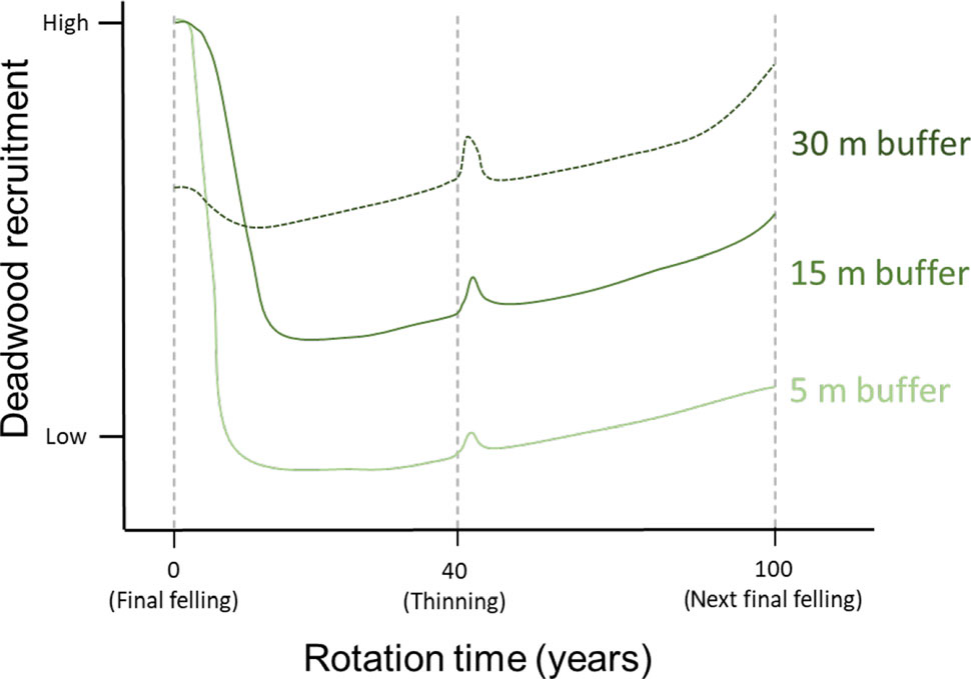
A conceptual model describing the recruitment of deadwood in riparian buffers over a typical rotation in northern Sweden (100 years). In the 5- and 15-m-wide buffers, the initial blown-down is high (based on the results of this study—solid lines), but it is likely that the 15-m-wide buffer will continue providing higher volumes of deadwood due to many trees remaining standing even after the initial wind-felling. In the 5 m buffer, most trees are blown down within the first decade, which decreases the deadwood recruitment in the next decades. In the 30 m buffers, we speculate (dashed line) that initial blowdowns would be less and occurring mostly at the outer edges of buffers. Furthermore, the 30 m buffer would keep providing deadwood continuously as the trees age. In all buffers, the provision of deadwood can also be affected by forestry operations, such as thinning, when the stem density of the adjacent stand decreases and wind exposure increases. In all buffers, provision of deadwood is hypothesized to also increase continuously because of new tree growth and potential mortality. Recruitment increases exponentially towards the end of the rotation as the trees that were left in the buffer (and did not blow down) are reaching 200 years. However, this incline is very low in the 5 m buffers, given that most of the trees from the previous rotation have been downed and the new trees are only 100 years old

In our two complimentary data sets, we found that deadwood recruitment into stream and riparian areas in managed forests of Sweden is largely affected by riparian buffer width. Narrow buffers can provide much needed deadwood when they inevitably blow down; however, there is a tipping point where the benefits of the increased volume of deadwood is likely outweighed by the other negative effects of so much windthrow (Fig. 6). Wider buffers (15 m) still provide high volumes of deadwood, while presumably provide some other functions, such as shading, better than 5 m buffers. Nevertheless, > 30-m-wide buffers have been recommended by many other studies from forests in the boreal zone as well as other regions when the goal is to prevent any changes to the aquatic-riparian diversity and functioning, including minimizing wind-felling. More research is needed to disentangle the complex relationships between buffer widths and ecological functions they should provide on the short as well as long term. It is possible that in some situations, 15 m buffers might suffice, while in others much wider buffers are necessary (Boradmeadow and Nisbet 2004). Importantly, buffers that do not blow down completely have the potential to keep providing deadwood continuously in the decades to come, thus, wider buffers are currently a safer strategy for riparian buffer management (Fig. 6). On the other hand, wider buffers represent a substantial monetary loss for forest owners, especially because today’s mature stands are composed of large crop trees (Norway spruce) that have been encouraged and managed for future harvest for decades. Those trees are both expensive to leave on site as well as susceptible to windthrows that would likely eventually be salvaged logged. We need to find solutions for buffer management that will allow extraction of a portion of these large crop trees from wide (30 m) riparian buffers, while minimizing management impacts on water. Hasselquist et al. (2021) provided a unique solution through targeted management during the entire rotation cycle of Swedish forest stands. Adopting their solutions would essentially require continuous cover forestry within riparian zones and encourage a more diverse, multi-story riparian forest at the time of final felling. This would also secure continuous recruitment of diverse deadwood of high quality, an aspect that is currently missing even from our heavily wind-felled buffers.

## Supplementary Information

Below is the link to the electronic supplementary material.

Supplementary file1 (PDF 596 kb)
