# Supplementary material for: Too much, too soon? Two Swedish case studies of short-term deadwood recruitment in riparian buffers
Source: Ambio. 2022 Oct 8;52(2):440–52. doi: 10.1007/s13280-022-01793-1 (PMC9755393; doi:10.1007/s13280-022-01793-1)
Supplement: Supplementary file 1 — Supplementary file1 (PDF 596 kb) [file 13280_2022_1793_MOESM1_ESM.pdf]

Abmio

Electronic Suppelemntary Material

This supplementary material has not been peer reviewed.

Title: **Too much, too soon? Two Swedish case studies of short-term deadwood recruitment in riparian buffers**

Authors: Lenka Kuglerová, Gustaf Nilsson, Eliza Maher Hasselquist

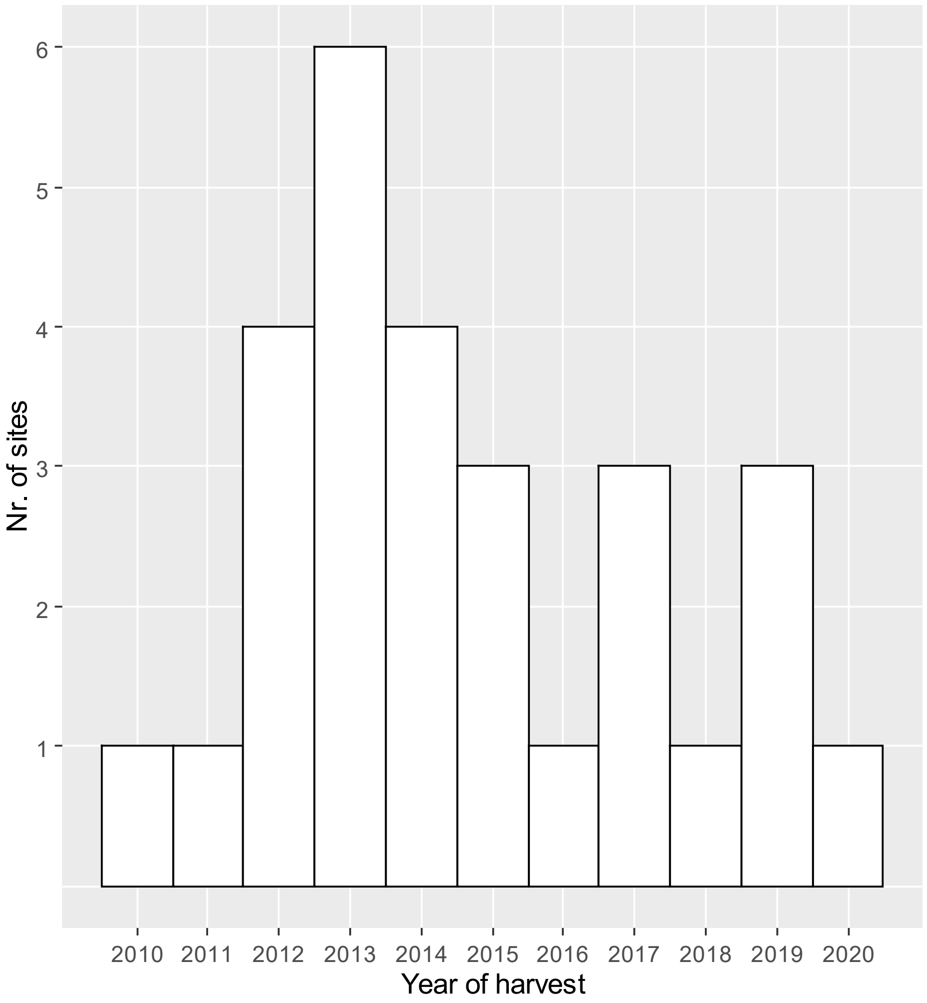

Figure S1. Histogram showing the number of sites in the regional data set and their year of harvest.

Table S1. The number of objects by species recorded at the six study reaches in the Trollberget experimental stream before and after harvest. The reaches received either 5 or 15 m wide riparian buffer.

	Species:	Downy birch		Scots pine		Norway spruce		Salix willow		unknown	
Reach	buffer	*before*	*after*	*before*	*after*	*before*	*after*	*before*	*after*	*before*	*after*
1 Narrow	5 m	18	25	0	1	7	37	0	0	16	5
1 Wide	15 m	4	11	0	1	1	56	1	0	14	5
2 Narrow	5 m	2	5	0	0	12	23	0	0	2	3
2 Wide	15 m	8	9	0	1	9	24	0	0	3	1
3 Narrow	5 m	8	12	1	1	5	8	0	0	8	5
3 Wide	15 m	31	22	0	1	10	22	2	3	4	5

Table S2. The number of objects recorded at the six study reaches in the Trollberget experimental stream before and after harvest. The reaches received either 5 or 15 m wide riparian buffer. The number of objects are sorted according to their placement in respect to the stream channel.

		bankful		bridge		in water	
Reach	buffer	*before*	*after*	*before*	*after*	*before*	*after*
1 Narrow	5 m	13	10	4	39	24	19
1 Wide	15 m	7	6	3	56	10	11
2 Narrow	5 m	2	6	10	22	4	3
2 Wide	15 m	2	7	8	19	10	9
3 Narrow	5 m	4	5	7	10	11	11
3 Wide	15 m	14	18	13	18	20	17
